# Discovery and identification of potential biomarkers of papillary thyroid carcinoma

**DOI:** 10.1186/1476-4598-8-79

**Published:** 2009-09-28

**Authors:** Yuxia Fan, Linan Shi, Qiuliang Liu, Rui Dong, Qian Zhang, Shaobo Yang, Yingzhong Fan, Heying Yang, Peng Wu, Jiekai Yu, Shu Zheng, Fuquan Yang, Jiaxiang Wang

**Affiliations:** 1 Department of Surgery, the First Affiliated Hospital, Zhengzhou University, Zhengzhou, Henan 450052, PR China; 2 Proteomic Platform, Institute of Biophysics, Chinese Academy of Sciences, Beijing 100101, PR China; 3 Institute of Cancer, the Second Affiliated Hospital, College of Medicine, Zhejiang University, Hangzhou, Zhejiang 310009, PR China

## Abstract

**Background:**

Thyroid carcinoma is the most common endocrine malignancy and a common cancer among the malignancies of head and neck. Noninvasive and convenient biomarkers for diagnosis of papillary thyroid carcinoma (PTC) as early as possible remain an urgent need. The aim of this study was to discover and identify potential protein biomarkers for PTC specifically.

**Methods:**

Two hundred and twenty four (224) serum samples with 108 PTC and 116 controls were randomly divided into a training set and a blind testing set. Serum proteomic profiles were analyzed using SELDI-TOF-MS. Candidate biomarkers were purified by HPLC, identified by LC-MS/MS and validated using ProteinChip immunoassays.

**Results:**

A total of 3 peaks (*m/z *with 9190, 6631 and 8697 Da) were screened out by support vector machine (SVM) to construct the classification model with high discriminatory power in the training set. The sensitivity and specificity of the model were 95.15% and 93.97% respectively in the blind testing set. The candidate biomarker with *m/z *of 9190 Da was found to be up-regulated in PTC patients, and was identified as haptoglobin alpha-1 chain. Another two candidate biomarkers (6631, 8697 Da) were found down-regulated in PTC and identified as apolipoprotein C-I and apolipoprotein C-III, respectively. In addition, the level of haptoglobin alpha-1 chain (9190 Da) progressively increased with the clinical stage I, II, III and IV, and the expression of apolipoprotein C-I and apolipoprotein C-III (6631, 8697 Da) gradually decreased in higher stages.

**Conclusion:**

We have identified a set of biomarkers that could discriminate PTC from non-cancer controls. An efficient strategy, including SELDI-TOF-MS analysis, HPLC purification, MALDI-TOF-MS trace and LC-MS/MS identification, has been proved successful.

## Background

Thyroid carcinoma is the most common endocrine malignancy and a common cancer among the malignancies of head and neck. It comprises 91.5% of all endocrine malignancies and 1% of all malignant diseases [1]. An estimated 33550 new cases are diagnosed annually in the United States and recent statistics shows the incidence of thyroid carcinoma has increased, especially in papillary thyroid carcinomas (PTC) [2]. PTC is the most common type, which accounts for 80% of all thyroid cancers [3]. Early accurate diagnosis and timely treatment are critical for improving long-term survival of PTC patients. Many diagnostic tools have been used for thyroid carcinoma, such as sonography, computed tomography, magnetic resonance imaging, cytological examination and fine-needle aspiration. Currently, although ultrasound-guided fine-needle aspiration biopsy is considered as the most effective test for distinguishing malignant from benign thyroid nodules, its sensitivity is approximately 93% and its specificity is 75% [4]. At the same time, researchers have been seeking valuable biomarkers for thyroid carcinoma diagnosis, such as galectin-3, fibronectin-1, CITED-1, HBME1, cytokeratin-19 and TPO, and so on. What is disappointing is that all these biomarkers either are lacking specificity to some degree, or have a poor positive predictive value [5-9]. To distinguish a malignant thyroid nodule from a benign lesion more accurately, the diagnostic test, however, still needs to be improved. Moreover, a noninvasive screening method for thyroid malignancy remains unavailable.

Recent advances in the proteomics study have introduced novel techniques for the screening of cancer biomarkers and improved early and accurate diagnosis of cancer diseases to a new horizon [10]. Surfaced enhanced laser desorption/ionization time of flight mass spectroscopy (SELDI-TOF-MS), which generates the protein fingerprint by MS, has been proved a powerful tool for potential biomarker discovery [11,12]. Recently, the SELDI-TOF-MS analysis has been successfully used to identify specific biomarkers for various cancers, such as ovarian cancer, prostate cancer, pancreatic cancer, colon cancer, breast cancer, *etc *[13-17]. In search of biomarkers for diagnosing PTC, a few pilot studies based on proteomics were conducted, in which SELDI-TOF-MS has been utilized [18,19]. However, no specific protein biomarkers have been identified and validated in those reports.

In this study, firstly, we used SELDI-TOF-MS technology to screen potential protein patterns specific for PTC and then purified the candidate protein biomarker peaks by HPLC, identified by LC-MS/MS and finally confirmed these biomarkers by ProteinChip Immunoassays. To the best of our knowledge, this is the first time that proteins biomarkers have been identified for PTC.

## Results

### Serum protein profiles and data processing

**Figure 1:**
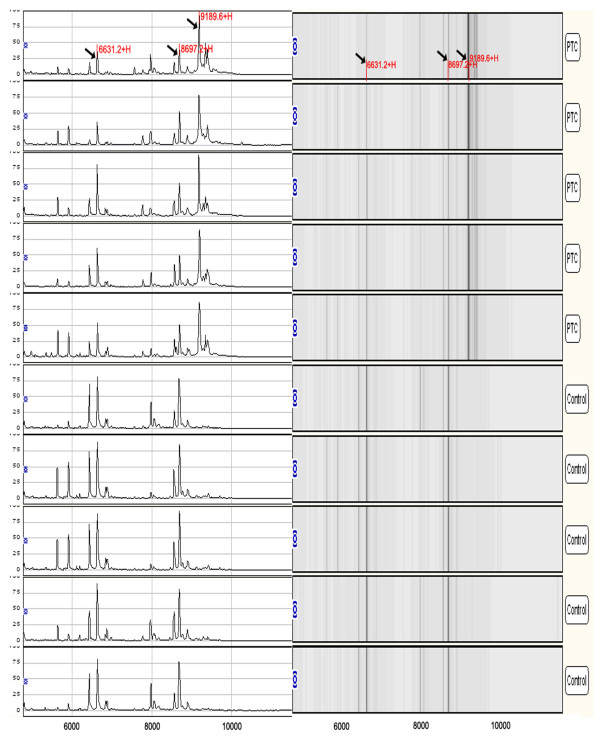
**A representative mapping of SELDI-TOF-MS analysis of sera from PTC patients and healthy controls**. Differentially expressed proteins with potential diagnostic significance are arrowed. Top-group denotes sera from patients with PTC, in which the protein with *m*/*z *of 9190 Da was over-expressed. Bottom-group denotes sera from healthy individuals, in which the proteins with *m*/*z *of 6631 and 8697 Da were up-regulated.

**Figure 2:**
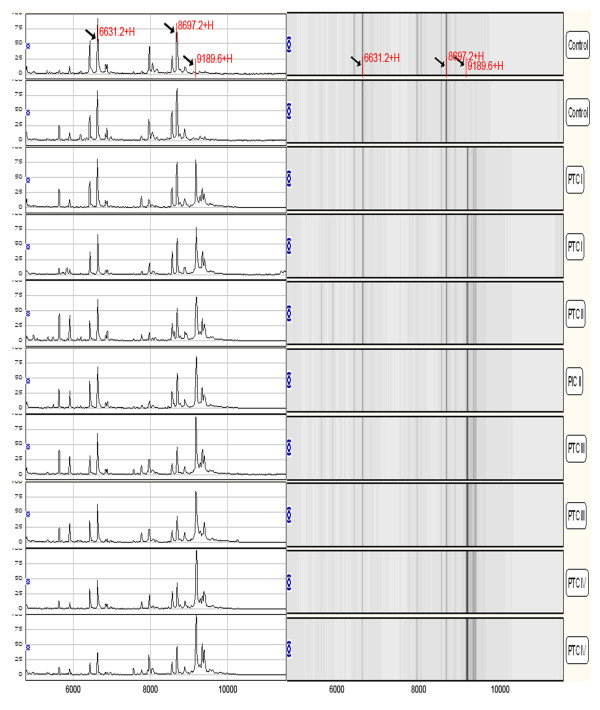
**A representative mapping of SELDI-TOF-MS analysis from different stages of PTC patients and non-cancer controls**. The level of the 9190 Da protein progressively increased with the clinical stage I, II, III and IV, and the expression of 6631 and 8697 Da proteins gradually decreased in higher stages.

Serum samples from the training set were analyzed and compared by SELDI-TOF-MS with WCX2 chip. All MS data were baseline subtracted and normalized using total ion current, and the peak clusters were generated by Biomarker Wizard software. After carrying out Wilcoxon rank sum tests to determine relative signal strength, 26 peaks with *p *value < 0.01 were obtained. Seven protein peaks were found up-regulated and 19 peaks were found down-regulated in PTC group (data not shown). From the random combination of protein peaks with remarkable variation, support vector machine (SVM) screened out the combined model with maximum Youden index of the predicted value, identifying 3 markers positioned at 9190, 6631 and 8697 respectively. In the PTC group, the 9190 Da protein was remarkably elevated while 6631 & 8697 Da proteins were significantly decreased (figure 1). The descriptive statistics of these 3 markers are shown in Table 1. In addition, the level of 9190 Da protein progressively increased with the clinical stage I, II, III and IV, and the expression of 6631, 8697 Da proteins gradually decreased in higher stages (figure 2). Combining 3 potential markers, using the method of leave-1-out for cross detection, the sensitivity of discriminating 60 PTC and 40 normal subjects was 98%, and its specificity was 97%.

**Table 1:** The relative peak intensity of three distinct protein spectra found in sera of PTC patients.

***m/z***	**Patients with PTC** **(mean ± SD)**	**Healthy Individuals** **(mean ± SD)**	***p***
9190	6853.82 ± 1585.23	282.46 ± 118. 09	0.000538
6631	2706.56 ± 578.17	4697.16 ± 989.85	0.001381
8697	3017.98 ± 600.28	4924.32 ± 1048.11	0.001672

### Protein peak validation

The remaining 48 PTC and 76 control serum samples (20 healthy controls and 56 patients with benign thyroid node) as a blind testing set, were analyzed to validate the accuracy and validity of the classification model derived from the training set. The descriptive statistics of the three markers in 48 PTC patients and 76 non-cancer controls are shown in Table 2. The classification model distinguished the PTC samples from controls with a sensitivity of 95.15%, specificity of 93.97%, and positive predictive value of 96.0%, respectively. The area under the receiver operating characteristics (ROC) curve of this model was 0.971.

**Table 2:** The descriptive statistics of these 3 markers in the blind testing set.

***m/z***	**Patients with PTC** **(mean ± SD)**	**Non-cancer Controls** **(mean ± SD)**	***p***
9190	6798.89 ± 1517.76	271.35 ± 102.04	0.000526
6631	2786.03 ± 596.11	4705.23 ± 991.45	0.001402
8697	3011.62 ± 598.13	4912.36 ± 1042.79	0.001669

### Purification and identification of candidate protein biomarkers

**Figure 3:**
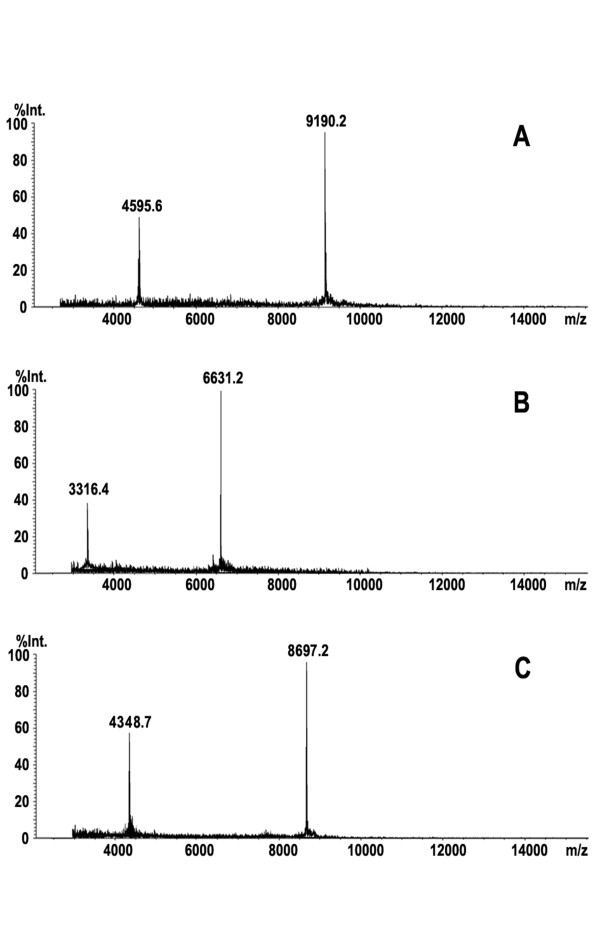
**MALDI-TOF-MS spectra of three purified potential protein markers**.

Serum samples from PTC patients were used for the purification of the up-regulated candidate protein biomarker (9190 Da), and serum samples from healthy controls were used for the purification of the two down-regulate proteins (6631, 8697 Da) using WCX SPE and C18 HPLC. Figure 3 shows the results of MALDI-TOF-MS analysis of the three purified candidate protein biomarkers.

**Figure 4:**
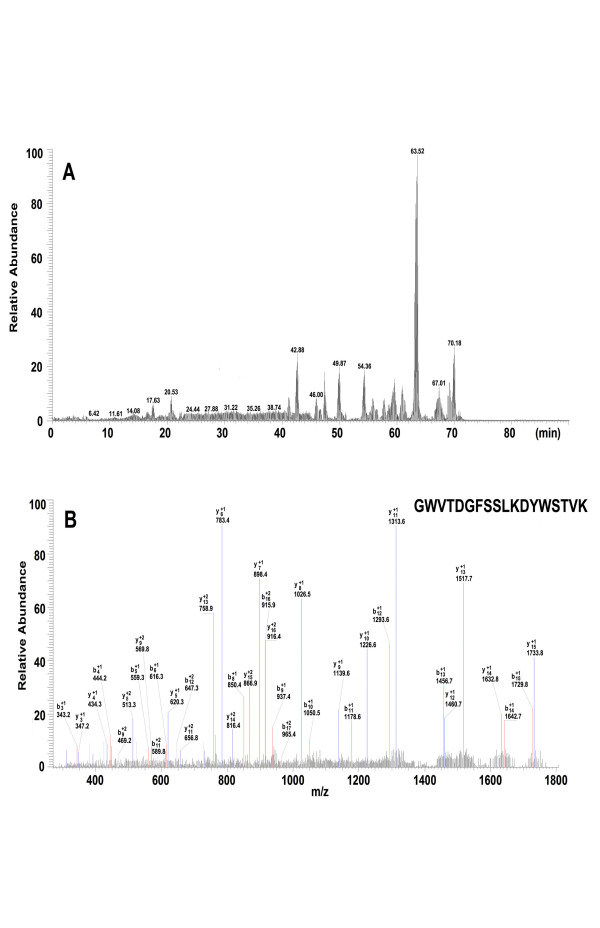
**Results of the identification of protein (8697 Da) by LC-MS/MS**. (A) Chromatogram of peptide mixture. (B) MS/MS spectrum of one peptide.

After digestion with modified trypsin, the peptide mixture was analyzed by nano-LC-MS/MS. Figure 4 shows the results of the LC-MS/MS chromatogram (A) and MS/MS spectrum of one identified peptide (B) from protein (8697 Da). Table 3 shows the results of identification of the three candidate protein biomarkers. They were haptoglobin alpha-1 chain (9190 Da) [NCBI:P00738], apolipoprotein C-I (6631 Da) [NCBI:P02654] and apolipoprotein C-III (8697 Da) [NCBI:CAA25233]. The whole sequence of the three candidate protein markers is given by combination of high sequence coverage and accurate molecular weight (MW) measurement using MALDI-TOF-MS.

**Table 3:** Identification of the three potential protein biomarkers with identified peptides and covered sequence.

***m/z***	**Protein Name**	**Peptides Identified**	Sequence*
9190	Haptoglobin α-chain	K.LRTEGDGVYTLNNEK.Q	addgcpkppeiahgyvehsv**ryqck nyyklrtegdgvytlnnekqwinka vgdklpeceavcgkpknpanpvq**
		R.YQCKNYYK.L	
		K.AVGDKLPECEAVCGK.P	
		K.QWINKAVGDK.L	
		R.TEGDGVYTLNNEK.Q	
		K.LPECEAYCGKPK.N	
		K.PKNPANPVQ.-	
6631	Apolipoprotein C-I	G.TPDVSSALDK.L	**tpdvssaldklkefgntledkarelis rikqselsakmrewfsetfqk**vkeklkids
		K.EFGNTLEDKAR.E	
		K.LKEFGNTLEDK.A	
		R.EWFSETFQK.V	
		G. TPDVSSALDKLK.A	
		K.MREWFSETFQK.V	
		K.ARELISRIK.Q	
8697	Apolipoprotein C-III	K.DALSSVQESQVAQQAR.G	seaedasllsfmqgymkhat**ktakdalssvq esqvaqqargwvtdgfsslkdywstvkd kfsefwdldpevrptsavaa**
		R.GWVTDGFSSLK.D	
		K.TAKDALSSVQESQVAQQAR.G	
		R.GWVTDGFSSLKDYWSTVK.D	
		K.DKFSEFWDLDPEVR.P	
		K.DKFSEFWDLDPEVRPTSAVAA.-	

*: Bold letters show the covered sequence by identified peptide

### Validation of three candidate protein biomarkers

**Figure 5:**
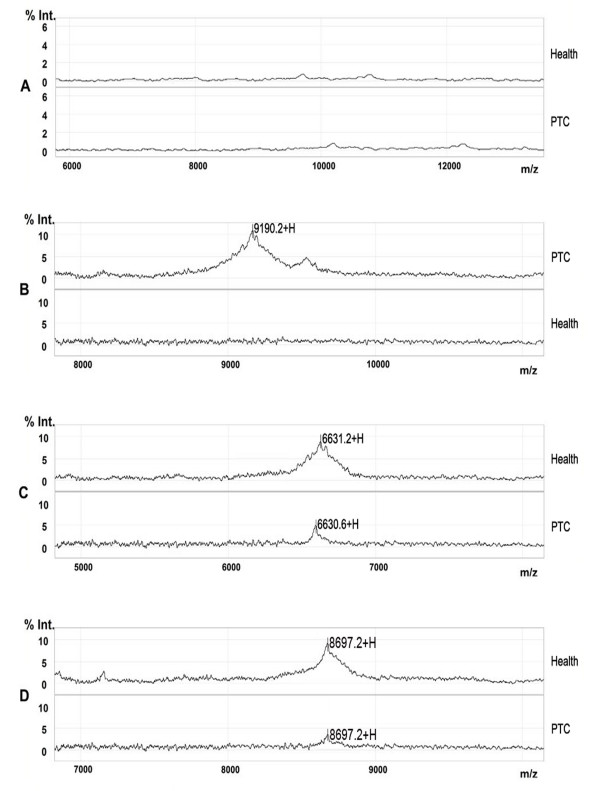
**Representative spectra from ProteinChip array with specific immobilized antibodies**. (A) Representative spectra of the negative control (nonspecific rabbit IgG). (B) Representative spectra from ProteinChip array with anti-haptoglobin alpha chain antibody. (C) Representative spectra from ProteinChip array with anti-apolipoprotein C-I. (D) Representative spectra from ProteinChip array with anti-apolipoprotein C-III.

A ProteinChip-array-based immunoassay (Ciphergen Biosystems) was used to specifically capture haptoglobin alpha-1 chain, apolipoprotein C-I and apolipoprotein C-III from crude serum samples and to confirm the significance of each marker. The anti-haptoglobin alpha-chain antibody specifically captured the previously identified 9190 Da protein. The anti-apolipoprotein C-I array was developed to capture apolipoprotein C-I (6631 Da) and the apolipoprotein C-III antibody against specifically captured apolipoprotein C-III (8697 Da). (Figure 5)

## Discussions

In this study, we obtained serum protein mass spectra from PTC patients and controls using SELDI-TOF-MS. Based on the serum proteomic profiles, we constructed a classification model to discriminate PTC patients from non-cancer controls. One of the challenges in the analysis of SELDI-TOF-MS-generated data is to reduce the false protein peaks, in which the discriminatory power is due to random variation [20]. To solve this problem in the data processing of this experiment, we eliminated noise by discrete wavelength, identified mass-charge peaks of specimens using the method of local extremum, and clustered mass-charge peaks by setting 10% as the minimum threshold. Wilcoxon rank sum test analysis assessed the relative importance of each peak in the discrimination of 2 kinds of specimen according to *P *values. Furthermore, SVM was employed in our experiment, which is a kind of classification technology proposed by Vapnik and others. In the model discrimination, the popularization, model selection, overfitting, latitude disaster, and other problems of the small specimen model have been solved successfully in SVM [21-23]. The procedures included randomly combining the remarkably different mass-charge peaks and inputting them into SVM, screening out the markers, building the discrimination model, and then using the method of leave one out to assess the model by means of cross verification. By combination among these procedures mentioned above, the popularization of the model building and the accuracy of the prediction were ensured. The classification model could discriminate patients with PTC from non-cancer controls with a sensitivity of 95.15% and a specificity of 93.97% in the blind testing set. The up-regulated candidate protein biomarker was identified as haptoglobin alpha-1 chain (9190 Da). Another two down-regulated candidate protein biomarkers (6631 and 8697 Da) were identified as apolipoprotein C-I and apolipoprotein C-III.

Among the proteins identified by LC-MS/MS, haptoglobin alpha-1 chain was significantly elevated in PTC patients and this protein may play a critical role in the development of PTC. Intact haptoglobin, composed of two different polypeptides (alpha and beta-chains), is an acute phase protein capable of binding haemoglobin and preventing iron loss [24]. It was reported that body iron could promote neoplastic cell growth and accumulate in cancer cells more than in normal cells [25]. Furthermore, a few other studies have demonstrated that there is a higher cancer risk in patients with larger iron stores than those with small iron stores [26]. Collectively, highly expressed haptoglobin results in a high hemoglobin/iron existence and raises the possibility of a causative involvement of iron-derived oxidative stress in the tumour development. Recently, there are several reports in the literature showing increased expression of haptoglobin in ovarian cancer, prostatic carcinoma and pancreatic cancer [27-29], and the level of haptoglobin alpha chain up-regulated in serum of breast cancer [30]. However, it is necessary to understand more clearly of the role of haptoglobin alpha-1 chain in the development of PTC through further studies of biological mechanisms of thyroid carcinoma.

Apolipoproteins (APOs) are lipid carriers and previous studies about APO mainly focused on lipoprotein metabolism. Recently, APOs have been reported to regulate many cellular function. For example, the protein kinase Akt can be elicited by APO C-I, which in turn promotes growth factor-mediated cell survival and block apoptosis [31]. In this study, the APO C-I is down-regulated in the serum of PTC patients, which indicates that APO C-I may be related to PTC. Thus, besides the function of APO C-I in lipid metabolism, additional function of APO C-I in cancerogenesis may also exist. However, the mechanism of how APO C-I is degraded in PTC is not very clear, and further research is required. APO C-III is primarily synthesized in the liver and in a minor degree in the small intestine. The regulatory elements that control both hepatic and intestinal transcription of the human APO C-III gene are localized between nucleotides -792 and -25 of the APO C-III promoter. The mechanism of why APO C-III was decreased in PTC may be as follows. Orphan members of the nuclear hormone receptor superfamily can bind to the hormone response elements (HREs), and strongly enhance or repress APO C-III promoter's activity. It was reported that a combination of RXRalpha and T3Rbeta could repress the APO C-III promoter's activity in the presence of T3 [32]. In addition, the thyroid stimulating hormone (TSH) level was significantly higher in PTC than in non-cancer control [33], correspondingly, T3 increased, thus the expression of APO C-III was possibly inhibited in PTC patients.

## Conclusion

In summary, we have identified a set of protein peaks that could discriminate PTC from non-cancer controls. From the protein peaks specific for PTC disease, we identified haptoglobin alpha-1 chain, apolipoprotein C-I and apolipoprotein C-III as potential proteomic biomarkers of PTC. Further studies with larger sample sizes will be needed to verify the specific protein markers. An efficient strategy, composed of SELDI-TOF-MS analysis, HPLC purification, MALDI-TOF-MS trace and LC-MS/MS identification has been proved very successful.

## Materials and methods

### Patients and serum samples

Serum samples were obtained from 224 individuals with informed consent in the Department of General Surgery, the First Affiliated Hospital of Zhengzhou University. These 224 individuals included 108 patients with PTC, 56 patients with benign thyroid node, and 60 healthy individuals. Patients with PTC had a median age of 43 years (ranging from 23 to 75 years, 27 men and 81 women), and the sera was obtained at the time of diagnosis. All 108 patients were distributed in 4 stages according to UICC. In stage I there were 85 patients, stage II, III & IV consisted of 12, 8 & 3 patients respectively. The diagnosis, stage and other information about these patients are listed in Table 4 and Table 5. Inclusion criteria for the study were patients with a confirmed diagnosis of PTC, the ability to provide written, informed consent, and without any previous treatment. Patients with benign thyroid node and healthy donors were defined as controls in this study. The benign thyroid node group and the healthy individual group were age- and gender-matched with the PTC group. Pathological diagnosis of all the PTC and benign thyroid nodes were confirmed independently by two pathologists. All serum samples were collected preoperatively in the morning before breakfast. The sera were left at room temperature for 1 h, centrifuged at 3000 rpm for 10 min, and then stored at -80°C.

**Table 4:** The diagnosis, staging and other information about PTC patients in the training set.

**Patient ID**	**Age (years)**	**Sex (F = female M = male)**	**Diagnosis**	**Stage**	**Tumor size (cm)**	**Cervial lymph node metastasis**	**Distant metastases**
Patients<45 years							
P38	23.01	F	PTC	I	0.5	None	NO
P78	29.98	F	PTC	I	1.0	None	NO
P65	32.12	F	PTC	I	1.0	None	NO
P81	36.45	F	PTC	I	0.8	None	NO
P36	38.50	F	PTC	I	0.5	None	NO
P43	39.56	F	PTC	I	1.0	None	NO
P16	40.05	F	PTC	I	0.8	None	NO
P44	40.11	M	PTC	I	1.0	Yes(VI lymph node)	NO
P37	40.25	F	PTC	I	1.6	None	NO
P5	40.36	F	PTC	I	1.0	None	NO
P18	40.50	M	PTC	I	0.6	None	NO
P80	40.81	M	PTC	I	0.7	None	NO
P93	40.90	F	PTC	I	1.0	None	NO
P67	41.00	F	PTC	I	1.0	None	NO
P46	41.08	F	PTC	I	1.5	None	NO
P9	41.10	M	PTC	I	1.8	Yes(VI lymph node)	NO
P35	41.26	M	PTC	I	1.0	None	NO
P47	41.38	F	PTC	I	0.8	None	NO
P13	41.44	F	PTC	I	1.0	None	NO
P6	41.52	M	PTC	I	0.6	None	NO
P17	41.55	F	PTC	I	1.5	Yes(VI lymph node)	NO
P40	41.62	F	PTC	I	0.8	None	NO
P64	41.71	F	PTC	I	0.8	None	NO
P111	41.89	M	PTC	I	1.3	None	NO
P69	42.14	M	PTC	I	1.2	None	NO
P2	42.25	F	PTC	I	1.6	Yes(VI lymph node)	NO
P12	42.36	F	PTC	I	0.8	None	NO
P7	42.56	F	PTC	I	0.8	None	NO
P4	42.73	M	PTC	I	0.6	None	NO
P49	43.20	F	PTC	I	1.2	None	NO
P8	43.66	M	PTC	I	1.5	Yes(VI lymph node)	NO
P70	43.80	F	PTC	I	0.7	None	NO
P42	44.10	F	PTC	I	1.5	None	NO
P114	44.30	M	PTC	I	0.8	None	NO
P83	44.45	F	PTC	I	1.3	None	NO
P99	44.60	M	PTC	I	0.6	None	NO
Patients>45 years							
P105	45.05	F	PTC	I	0.8	None	NO
P71	45.58	M	PTC	II	2.5	None	NO
P107	45.75	M	PTC	I	0.7	None	NO
P60	45.81	F	PTC	II	2.5	None	NO
P10	46.48	F	PTC	I	0.8	None	NO
P104	46.40	F	PTC	II	2.3	None	NO
P14	46.90	F	PTC	II	2.5	None	NO
P52	47.10	F	PTC	I	0.7	None	NO
P11	47.64	M	PTC	III	3.5	Yes(VI lymph node)	NO
P88	47.89	F	PTC	I	0.6	None	NO
P22	48.50	F	PTC	I	0.8	None	NO
P73	48.81	M	PTC	I	0.7	None	NO
P87	49.56	F	PTC	III	4.5	Yes(VI lymph node)	NO
P100	49.89	F	PTC	II	2.8	None	NO
P25	50.25	F	PTC	III	3.8	Yes(VI lymph node)	NO
P85	50.89	F	PTC	I	0.7	None	NO
P101	57.14	M	PTC	II	2.5	None	NO
P32	58.21	F	PTC	II	2.3	None	NO
P103	60.10	F	PTC	I	0.8	None	NO
P53	61.12	F	PTC	II	2.5	None	NO
P23	65.84	F	PTC	IV	4.0	Yes(hibateral)	Yes(pulmonary metastasis)
P54	68.19	F	PTC	IV	3.5	Yes(mediastinal)	Yes(osseous metastasis)
P24	72.31	F	PTC	III	3.6	None	NO
P63	74.98	F	PTC	IV	3.5	Yes(hibateral)	Yes(pulmonary metastasis)

**Table 5:** The diagnosis, staging and other information about PTC patients in the blind testing set.

**Patient ID**	**Age (years)**	**Sex (F = female M = male)**	**Diagnosis**	**Stage**	**Tumor size (cm)**	**Cervial lymph node metastasis**	**Distant metastases**
Patients<45 years							
P57	25.50	F	PTC	I	0.6	None	NO
P64	41.71	F	PTC	I	0.8	None	NO
P15	31.25	F	PTC	I	0.5	None	NO
P19	34.16	F	PTC	I	0.6	None	NO
P79	37.71	F	PTC	I	0.7	None	NO
P91	38.63	F	PTC	I	1.2	Yes (VI lymph node)	NO
P110	39.80	F	PTC	I	1.0	None	NO
P34	40.20	F	PTC	I	0.6	None	NO
P66	40.28	M	PTC	I	0.7	None	NO
P92	40.42	F	PTC	I	1.0	None	NO
P109	40.56	F	PTC	I	0.5	None	NO
P1	40.85	F	PTC	I	0.7	None	NO
P94	41.15	F	PTC	I	0.7	None	NO
P108	41.30	F	PTC	I	1.6	Yes (VI lymph node)	NO
P95	41.39	M	PTC	I	1.2	None	NO
P68	41.46	F	PTC	I	0.8	None	NO
P106	41.58	M	PTC	I	1.5	None	NO
P96	41.66	F	PTC	I	0.7	None	NO
P30	41.82	F	PTC	I	1.0	None	NO
P39	42.05	F	PTC	I	1.0	None	NO
P41	42.20	F	PTC	I	1.8	None	NO
P89	42.31	M	PTC	I	0.7	None	NO
P48	42.45	M	PTC	I	1.2	None	NO
P98	42.68	F	PTC	I	1.5	None	NO
P113	42.80	F	PTC	I	0.9	None	NO
P97	43.40	F	PTC	I	1.0	None	NO
P29	43.72	F	PTC	I	1.6	Yes (VI lymph node)	NO
P90	44.26	M	PTC	I	0.8	None	NO
P50	44.54	F	PTC	I	0.7	None	NO
P56	44.98	F	PTC	I	0.7	None	NO
Patients>45 years							
P28	45.52	F	PTC	I	0.8	None	NO
P27	45.66	F	PTC	II	2.8	None	NO
P51	46.05	F	PTC	I	0.6	None	NO
P55	46.62	M	PTC	I	0.8	None	NO
P72	46.85	F	PTC	II	2.8	None	NO
P84	47.25	F	PTC	I	0.6	None	NO
P33	48.30	F	PTC	I	0.8	None	NO
P102	48.73	F	PTC	III	3.8	Yes (VI lymph node)	NO
P31	49.14	F	PTC	I	0.6	None	NO
P20	49.60	F	PTC	I	0.9	None	NO
P61	49.91	M	PTC	I	0.8	None	NO
P77	50.40	F	PTC	I	0.8	None	NO
P21	52.30	F	PTC	I	0.9	None	NO
P74	54.56	M	PTC	III	3.5	Yes (VI lymph node)	NO
P62	56.05	F	PTC	III	3.5	Yes (VI lymph node)	NO
P86	62.13	M	PTC	II	2.8	None	NO
P75	67.40	F	PTC	II	2.5	None	NO
P76	68.56	F	PTC	III	3.8	None	NO

### Reagents and instruments

Sinapinic acid (SA) was purchased from Fluka (USA). ProteinChip Biosystems (Ciphergen PBS II plus SELDI-TOFMS) and WCX2 chip were purchased from Ciphergen Biosystems (USA). All other SELDI-TOF-MS related reagents were acquired from Sigma (USA). Ziptip C18 was purchased from Millipore (USA). Trypsase was purchased from Promega (USA). IAM was purchased from AppliChem (GER). DTT was purchased from BIO-RAD (GER). MALDI-TOF-MS was purchased from Kratos Analytical Co (UK) and HPLC was purchased from Shimadzu (JPN). LC-MS/MS was purchased from Thermo Electron Corporation (USA).

### SELDI-TOF-MS analysis of serum protein profiles

Protein profiling of serum samples was determined by SELDI-TOF-MS using the WCX2 (weak cation exchange) Proteinchip arrays (Ciphergen Biosystems, USA). Frozen serum samples were defrosted on ice and spun at 10 000 rpm for 5 min at 4°C. Each serum sample (10 μl) was denatured by addition of 20 μl of U9 buffer (9 M urea, 2% CHAPS, 50 mM Tris-HCI, 1% DTT, PH 9.0) and vortexed at 4°C for 30 min. Each sample was then diluted in 108 μl of low stringency buffer (0.1 M sodium acetate, PH 4.0) and 100 μl of each diluted serum sample was hybridized with WCX2 proteinchip arrays, which was held by a bioprocessor (Ciphergen Biosystems) and preactivated twice with 150 μl low stringency buffer at room temperature for 5 min. The diluted serum sample was added on the surface of the WCX2 chip for 60 min at room temperature. Each spot was then washed three times with appropriate buffers of various PHs and ionic strengths to eliminate non-adsorbed proteins. After drying the array surface in the air, 1 μl saturated sinapinic acid (SA) matrix in 50% ACN and 0.5% TFA was applied and allowed to dry. MS analysis was performed on a PBS-II ProteinChip reader (Ciphergen Biosystems). Mass peak detection was analyzed using ProteinChip Biomarker Software version 3.1 (Ciphergen Biosystems). The mass spectra of the proteins were generated using an average of 140 laser shots at a laser intensity of 170 arbitrary units and detector sensitivity was set at 6. For data acquisition of low-molecular- weight proteins, the optimize detection mass range was set from 2 to 20 kDa for all study sample profiles. The instrument was calibrated by the All-in-one peptide molecular mass standard (Ciphergen Biosystems).

### Bioinformatics and biostatistics

Patients with PTC were split into a training set and a blind testing set. Sixty samples of PTC patients (45 stage I, 8 stage II, 4 stage III, 3 stage IV) and 40 healthy controls were selected for a training sample set randomly. To evaluate the accuracy and validity of the classification model, the remaining 48 samples of PTC patients (40 stage I, 4 stage II, 4 stage III) and 76 controls (20 healthy controls and 56 patients with benign thyroid node) were selected for a blind testing set. (Table 6).

**Table 6:** Study population used in SELDI experiment

	**PTC Patients**	**Controls**	**Total**
Training set	60	40 h*	100
Testing set	48	76(20 h*+56p**)	124

Total	108	116	224

h*: Healthy controls

p**: Patients with benign thyroid node

The first step of data analysis was to use the undecimated discrete wavelet transform (UDWT) method to denoise the signals. Secondly, the spectra were subjected to baseline correction by aliging with a monotone local minimum curve and mass calibration. The proteomic peaks were detected and quantified by an algorithm that takes the maximal height of every denoised, baseline-corrected, and calibrated mass spectrum into account. Thirdly, the peaks were filtered to maintain a S/N of more than three. The S/N of a peak is the ratio of the height of the peak above the baseline to the wavelet-defined noise. Finally, to match peaks across spectra, we pooled the detected peaks if the relative difference in their mass sizes was not more than 0.3%. The minimal percentage of each peak, appearing in all the spectra, is specified to ten. The matched peak across spectra is defined as a peak cluster. If a spectrum does not have a peak within a given cluster, the maximal height within the cluster will be assigned to its peak value. The normalization was performed only with the identified peak clusters.

To distinguish between data of different groups, we used a nonlinear SVM classifier, originally developed by Vladimir Vapnik, with a radial-based function kernel, a parameter Gamma of 0.6, and a cost of the constrain violation of 19. The leave-one-out crossing validation approach was applied to estimate the accuracy of this classifier. The capability of each peak in distinguishing data of different groups was estimated by the *p *value of Wilcoxson *T*-test. The *p *value was set at 0.01 to be statistically significant. The remaining 48 samples of PTC patients and 76 controls (20 healthy controls and 56 patients with benign thyroid node), were analyzed to test the classification model. PTC and control samples were then discriminated based on their proteomic profile characteristics. The sensitivity was defined as the probability of predicting PTC cases, the specificity was defined as the probability of predicting control samples. The positive predictive value was defined as the probability of PTC if a test result was positive.

### Serum fractionation

Serum samples both from healthy controls and PTC patients were selected for the purification of the three candidate protein biomarkers. The serum sample was mixed with U9 buffer (1:2, v/v) and incubated for 30 min at room temperature. The sample was then diluted in 5 mL WCX binding buffer (50 mM NaAc, pH 4.0) and loaded to the CM Ceramic Hyper D WCX SPE column (6 × 10 mm, Pall Life science, USA). After washing with 2 mL of WCX binding buffer, the column was eluted with 5 ml of eluting buffer (2 M NaCl, 50 mM NaAc, pH 4.0) at a flow rate of 0.5 ml/min. The eluted fraction was further purified using HPLC.

### Purification of candidate protein markers using HPLC

HPLC separation was performed using SCL-10AVP (Shimadzu, Japan) with a Sunchrom C18 column (250 × 4.6 mm, 5 μm particle size, 300 Å) (The Great Eur-Asia Sci-Tech Development Co. Ltd, Beijing, China) and a C18 guard column (10 × 3 mm, Shimadzu, Japan). The mobile phase consisted of solvent A (5% ACN, 0.1% TFA) and solvent B (90% ACN, 0.1% TFA). The HPLC separation was achieved with a linear solvent gradient: 100% A (0 min)-15% B (15 min)-65% B (65 min)-100% B (100 min) at a flow-rate of 0.5 ml/min. The eluate was detected at multiple wavelengths of 214, 254, 280 nm. Each peak fraction was collected and concentrated using SpeedVac, and then analyzed using AXIMA-CFRTM plus MALDI-TOF mass spectrometer (Shimadzu/Kratos, Manchester, UK) in linear mode to trace the candidate protein biomarkers with α-cyano-4-hydrorycinnamic acid (CHCA) as matrix.

### Identification of candidate protein biomarkers by LC-MS/MS

In-solution digestion of each concentrated fraction, which contains one candidate protein biomarker, was performed with a standard protocol. Briefly, each fraction was dissolved in 25 mM NH_4_HCO_3_, and reduced with 10 mM DTT for 1 hour, alkylated by 40 mM iodoacetamide in the dark for 45 min at room temperature, and then 40 mM DTT was added to quench the iodoacetamide for 30 min at room temperature. Then proteins were proteolysed with 20 ng of modified trypsin (Promega, Madison, WI) in 25 mM NH_4_HCO_3 _overnight at 37°C. The supernatant was collected and peptides were further extracted in 0.1% acetic acid and 60% acetonitrile. Peptide extracts were vacuumdried and resuspended in 20 μl of water for mass analysis. Protein digests obtained above were loaded onto a home-made C18 column (100 mm × 100 μm) packed with Sunchron packing material (SP-120-3-ODS-A, 3 μm) and followed with nano-LC-ESI-MS/MS analysis. The LTQ mass spectrometer was operated in a data-dependent mode, in which the initial MS scan recorded the *m/z *ratios of ions over the mass range from 400-2000 Da firstly, and then the five most abundant ions were automatically selected for subsequent collision-activated dissociation. All MS/MS data were searched against a human protein database downloaded from NCBI using the SEQUEST program (Thermo, USA).

### Confirmation of candidate protein biomarkers using ProteinChip Immunoassays

To confirm the identity of the candidate protein biomarkers, all samples from the initial experiments were reanalyzed by using ProteinChip immunoassays (Ciphergen Biosystems). Specific antibody arrays were prepared by covalently coupling the appropriate antibodies to preactivated ProteinChip arrays (Ciphergen Biosystems). Antibodies (anti-human haptoglobin alpha-chain, ms1750rb-h; anti-human apolipoprotein C-I, ab9106; anti-human apolipoprotein C-III, mab002-74/3) were covalently coupled to PS20 arrays, respectively. After blocking with BSA and washing to remove uncoupled antibodies, antibody-coated spots were incubated with 1.5 μL of serum samples and 3 μL of binding buffer (0.1 M Na_3_PO_4_, 0.5 M urea, 0.5% CHAPS, pH 7.2) for 90 min. Spots were then washed with PBST (0.5% Triton X-100), PBS and deionized water twice respectively before drying. SELDI-TOF-MS analysis was performed on a PBS-II ProteinChip reader with CHCA as matrix.

## Abbreviations

**PTC**: papillary thyroid carcinoma; **SELDI-TOF-MS**: surface-enhanced laser desorption/ionization time-of-flight mass spectroscopy; ***m/z***: mass to charge; **HPLC**: high Performance Liquid Chromatography; **LC-MS/MS**: liquid chromatography tandem mass spectrometry; **MALDI-TOF-MS**: matrix-assisted laser desorption/ionization time-of-flight mass spectroscopy; **WCX**: weak cation exchange; **SVM**: support vector machine; **S/N**: signal to noise ratio; **NCBI**: national center for biotechnology information.

## Competing interests

The authors declare that they have no competing interests.

## Authors' contributions

WJX and YFQ designed the study. FYX was responsible for laboratory studies and drafted the manuscript. LQL, DR and ZQ carried out the SELDI-TOF experiments. SLN, WP and YSB participated in the purification and identification of all the biomarkers. YJK carried out the SELDI-TOF data analysis. FYZ and YHY participated in data analysis. ZS participated in the design of the study and helped to draft the manuscript. All authors read and approved the final manuscript.
